# The Epidemiology and Clinical Features of Non-Keratitis *Acanthamoeba* Infections in the United States, 1956–2020

**DOI:** 10.1093/ofid/ofac682

**Published:** 2023-01-12

**Authors:** Julia C Haston, Kevin O’Laughlin, Kelsey Matteson, Shantanu Roy, Yvonne Qvarnstrom, Ibne K M Ali, Jennifer R Cope

**Affiliations:** Epidemic Intelligence Service, Centers for Disease Control and Prevention, Atlanta, Georgia, USA; Waterborne Disease Prevention Branch, Division of Foodborne, Waterborne, and Environmental Diseases, National Center for Emerging and Zoonotic Diseases, Centers for Disease Control and Prevention, Atlanta, Georgia, USA; Waterborne Disease Prevention Branch, Division of Foodborne, Waterborne, and Environmental Diseases, National Center for Emerging and Zoonotic Diseases, Centers for Disease Control and Prevention, Atlanta, Georgia, USA; Waterborne Disease Prevention Branch, Division of Foodborne, Waterborne, and Environmental Diseases, National Center for Emerging and Zoonotic Diseases, Centers for Disease Control and Prevention, Atlanta, Georgia, USA; Epidemiology Elective Program, Centers for Disease Control and Prevention, Atlanta, Georgia, USA; Waterborne Disease Prevention Branch, Division of Foodborne, Waterborne, and Environmental Diseases, National Center for Emerging and Zoonotic Diseases, Centers for Disease Control and Prevention, Atlanta, Georgia, USA; Parasitic Diseases Branch, Division of Parasitic Diseases and Malaria, Center for Global Health, Centers for Disease Control and Prevention, Atlanta, Georgia, USA; Waterborne Disease Prevention Branch, Division of Foodborne, Waterborne, and Environmental Diseases, National Center for Emerging and Zoonotic Diseases, Centers for Disease Control and Prevention, Atlanta, Georgia, USA; Waterborne Disease Prevention Branch, Division of Foodborne, Waterborne, and Environmental Diseases, National Center for Emerging and Zoonotic Diseases, Centers for Disease Control and Prevention, Atlanta, Georgia, USA

**Keywords:** *Acanthamoeba*, encephalitis, free-living ameba, immunocompromised

## Abstract

**Background:**

*Acanthamoeba* is a free-living ameba that can cause severe disease affecting the central nervous system, skin, sinuses, and other organs, particularly in immunocompromised individuals. These rare but severe infections are often fatal, yet incompletely described.

**Methods:**

Cases included were either reported to the Centers for Disease Control and Prevention (CDC) Free-Living Ameba program or published in scientific literature. Characteristics of all patients in the United States with laboratory-confirmed non-keratitis *Acanthamoeba* infections were described using descriptive statistics, and associations with survival were determined using χ^2^ and Fisher exact tests.

**Results:**

Of 173 patients identified, 71% were male and the median age was 44 years (range, 0–87 years). Of these, 26 (15%) survived. Most patients (88%) had at least 1 immunocompromising condition, most commonly human immunodeficiency virus (39%), cancer (28%), and solid organ or hematopoietic stem cell transplant (28%). Granulomatous amebic encephalitis (GAE) was the most common disease presentation (71%). Skin (46%), sinuses (29%), lungs (13%), and bone (6%) were also involved. Nearly half of patients (47%) had involvement of >1 organ system. Survival was less frequent among those with GAE (3%, *P* < .001) compared with cutaneous disease, rhinosinusitis, or multiorgan disease not including GAE. Of 7 who received the currently recommended treatment regimen, 5 (71%) survived.

**Conclusions:**

Non-keratitis *Acanthamoeba* infections occur primarily in immunocompromised individuals and are usually fatal. Survival may be associated with disease presentation and treatment. Providers who care for at-risk patients should be aware of the various disease manifestations to improve early recognition and treatment.

## Teaser

*Acanthamoeba* can cause rare but often fatal infections of the central nervous system, skin, sinuses, and other organ systems, especially among immunocompromised people. Recognizing the various disease manifestations and risk factors may improve early diagnosis and patient outcomes.

*Acanthamoeba*, along with *Naegleria fowleri* and *Balamuthia mandrillaris*, is a pathogenic free-living ameba (FLA) that can cause life-threatening disease in humans. Although *Acanthamoeba* is most known for causing isolated keratitis in healthy persons, invasive disease involving the skin, sinuses, central nervous system (CNS), or other organs can occur, especially in immunocompromised hosts [1]. *Acanthamoeba* infections have been described worldwide, and the organism is ubiquitous in the environment. It has been isolated from soil and water, and it was detected in 51% of United States (US) household tap water and environmental samples in one study [1–3]. Although most people are likely exposed to *Acanthamoeba* regularly, disease is rare in humans, with only a few reports of invasive disease each year in the US.

The first definitive report of *Acanthamoeba* causing granulomatous amebic encephalitis (GAE), an infection of the CNS, was described in 1972, though retrospective analysis determined the first known case to have occurred in 1956 [2, 4–6]. Cases were infrequently identified in subsequent years among immunosuppressed patients, but it was not until human immunodeficiency virus (HIV) became prevalent in the 1980s that non-keratitis *Acanthamoeba* disease was recognized as a primarily opportunistic pathogen [7]. In contrast to other FLA, *Acanthamoeba* nearly exclusively causes invasive disease in patients with impaired immune systems [1, 8–11]. Patients with immunosuppression due to uncontrolled HIV, malignancy, history of organ transplantation, diabetes mellitus, or immunosuppressive therapy are particularly susceptible as the ameba is unable to evade components of the intact human immune system [7, 10, 12–16].

The most common presentation of non-keratitis *Acanthamoeba* infections is GAE, which typically presents with insidious onset of encephalitis [1, 17]. Although GAE was the earliest recognized manifestation of *Acanthamoeba* infection and remains the most frequently described presentation, the ability to infect many other organ systems differentiates *Acanthamoeba* from other FLA. While *Naegleria fowleri* exclusively causes primary amebic meningoencephalitis and *Balamuthia mandrillaris* causes GAE with occasional skin involvement, *Acanthamoeba* can infect many different parts of the body, either in concurrence with GAE or independently [8, 9]. The most commonly described non-GAE presentations include cutaneous disease and rhinosinusitis [1, 18–20]. Other disease manifestations such as osteomyelitis, pneumonia, and disseminated disease involving multiple organs have also been reported [21–26]. Patient characteristics associated with disease presentation or severity have not been fully described.

Non-keratitis *Acanthamoeba* infections are often fatal. Because survival is uncommon, risk factors for mortality and factors associated with survival have not yet been identified. Currently, a multidrug regimen is recommended by the Centers for Disease Control and Prevention (CDC) for treatment of invasive *Acanthamoeba* infections; however, this recommendation is largely based on in vitro drug effectiveness studies and survivor case reports. Providing evidence-based treatment recommendations is challenging because few infections are diagnosed each year, many of which are diagnosed during postmortem examinations. Understanding factors associated with survival could help identify those at highest risk of death and could ultimately determine interventions that could improve outcomes.

## METHODS

**Figure 1.**
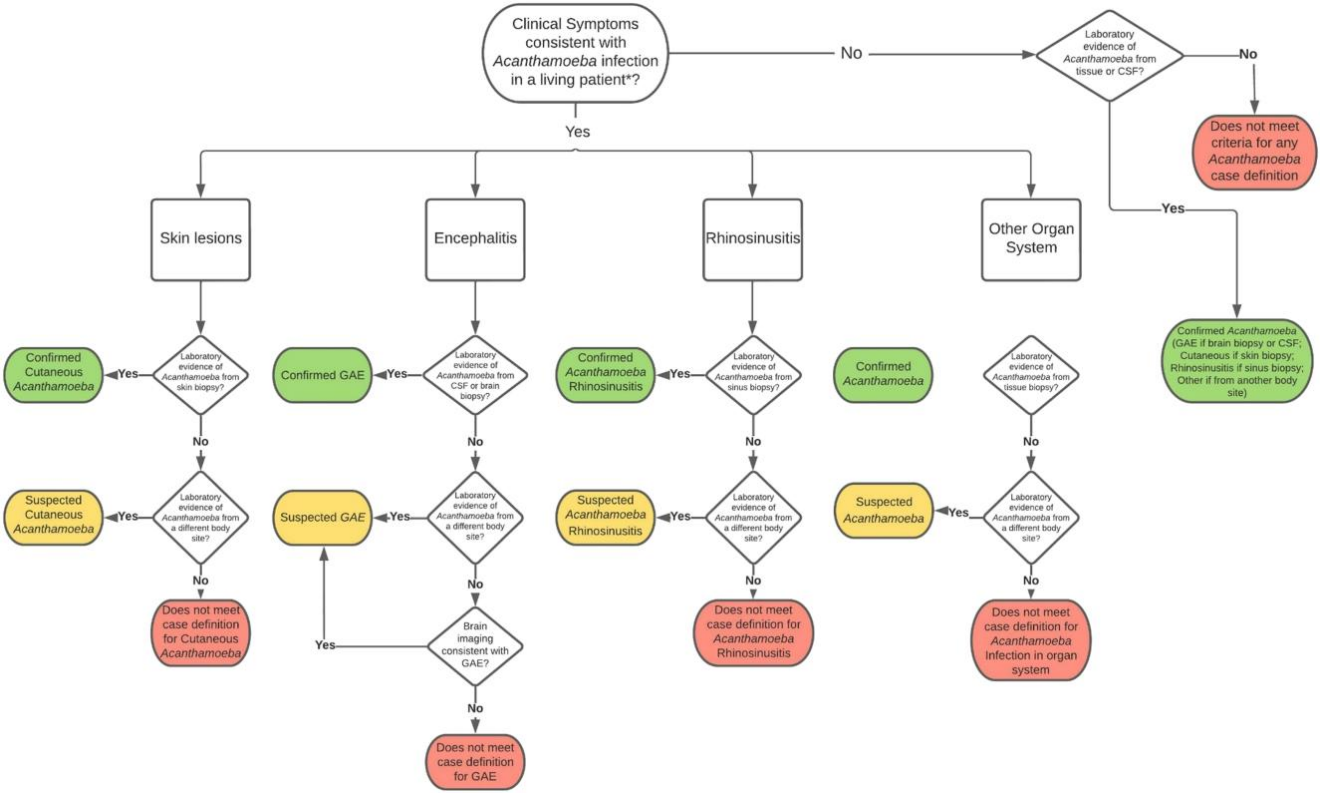
Case classification algorithm for assigning disease classification for patients with laboratory-confirmed non-keratitis *Acanthamoeba* infection. *As determined or suspected by healthcare provider. Abbreviations: CSF, cerebrospinal fluid; GAE, granulomatous amebic encephalitis.

The CDC Free-Living Ameba database was used for this analysis. Patients were either reported to CDC by clinicians or identified through published case reports in scientific literature. All reported US cases of non-keratitis *Acanthamoeba* infection with laboratory confirmation (ie, those that met the national case definition) from the first identified case through December 2020 were included [27]. Techniques for laboratory confirmation included microscopy, polymerase chain reaction (PCR), DNA sequencing, and immunohistochemical staining. The disease presentations for each case were determined to be “confirmed” or “suspected” according to the case classification algorithm (Figure 1).

Case year was the calendar year during which symptoms began. Geographic distribution was defined as state of residence, or if unavailable, state in which treatment was provided. A patient was considered to have survived if they were known to have recovered from their illness and remained alive when the case was reported, during follow-up, or at time of case publication.

Descriptive analyses were performed. Associations between patient characteristics and survival were assessed using χ^2^ tests of proportion, Fisher exact tests, and logistic regression. Associations between receiving a medication and survival were assessed among those with antemortem diagnoses only. Significance was defined as *P* value <.05. SAS version 9.4 software was used for all analyses.

This activity was reviewed by CDC and was conducted consistent with applicable federal law and CDC policy (see, eg, 45 Code of Federal Regulations [CFR] part 46, 21 CFR part 56; 42 US Code [USC] §241(d); 5 USC §552a; 44 USC §3501 et seq).

## RESULTS

**Figure 2.**
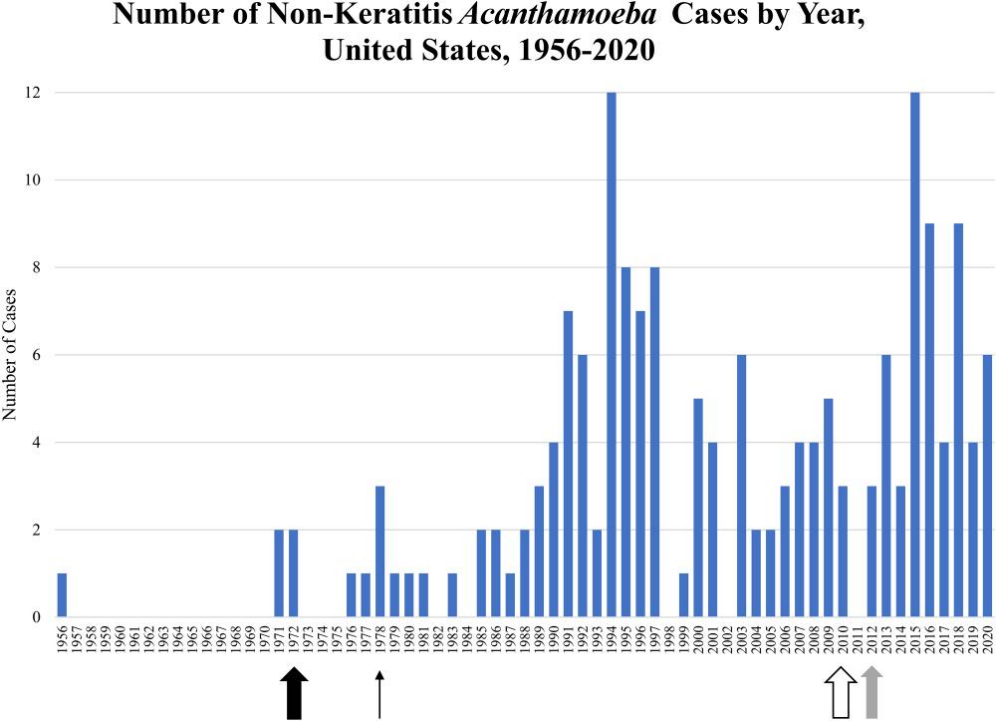
Number of non-keratitis *Acanthamoeba* cases by year, United States, 1956–2020.#Thick arrow denotes the year *Acanthamoeba* was first diagnosed in a human (1972). Prior cases were diagnosed retrospectively. Thin arrow denotes establishment of the Centers for Disease Control and Prevention (CDC) free-living and intestinal ameba laboratory (1978). Outlined arrow denotes establishment of CDC free-living ameba clinical consultation service (2010). Gray arrow denotes establishment of a national case definition for *Acanthamoeba* disease (excluding keratitis) (2012).

**Figure 3.**
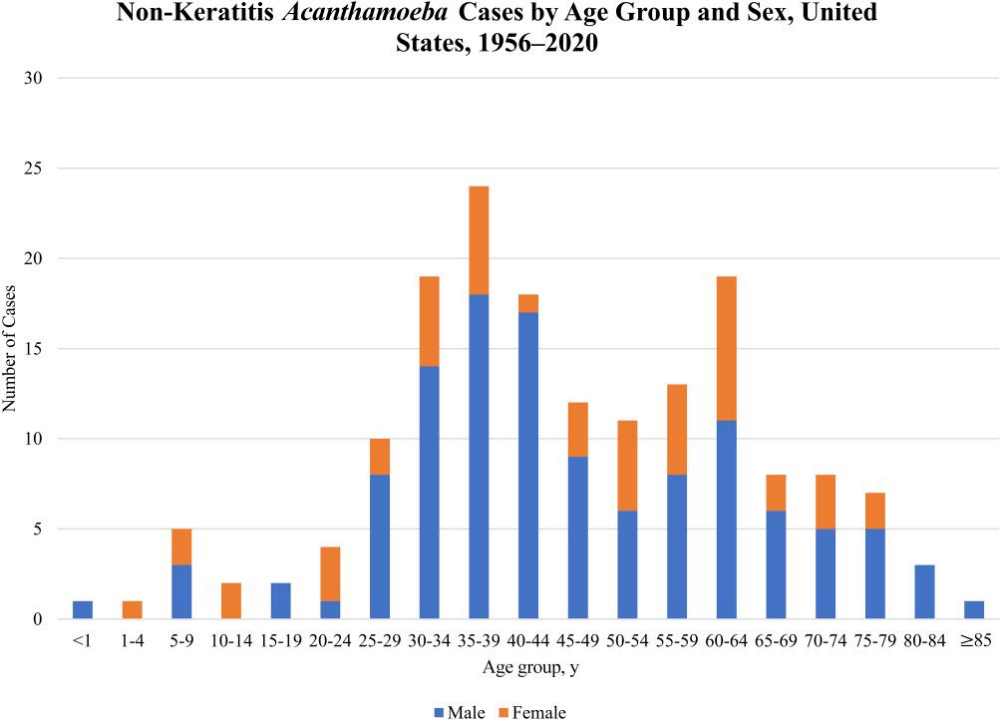
Non-keratitis *Acanthamoeba* cases by age group and sex, United States, 1956–2020.#Age and sex information was available for 168 patients.

**Figure 4.**
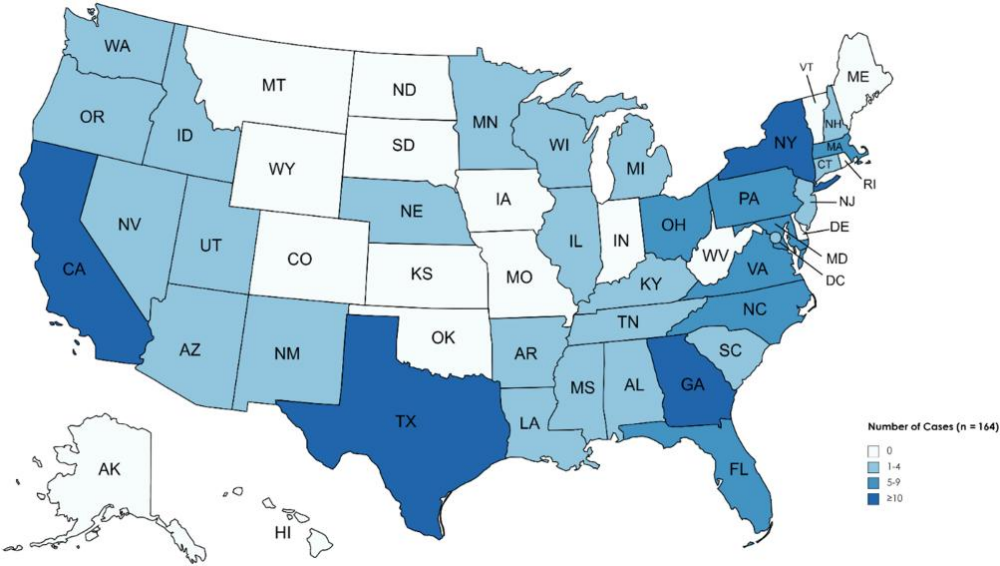
Geographic distribution of non-keratitis *Acanthamoeba* cases, United States, 1956–2020. State of residence was used for geographic classification; state of treatment was used for cases where state of residence was unavailable. State for 9 cases remains unknown.

In total, 173 patients were diagnosed with non-keratitis *Acanthamoeba* infections between 1956 and 2020 in the US. The median number of cases reported annually since 1970 was 3 per year (range, 0–12; Figure 2). Patient ages ranged from 8 months to 87 years; 80% of patients with a known age were between 25 and 69 years (Table 1, Figure 3). Eleven patients (7%) with a known age were <18 years of age. The majority (71%) of patients were male. For the 79 patients with reported race, 66% were White, 27% were Black, and 8% were of another race. Of the 40 patients with reported ethnicity, 43% were Hispanic (Table 1). *Acanthamoeba* cases were reported in 34 US states and districts (Figure 4). Of the 164 cases with known state of residence or treatment, 41% occurred in 4 states: California (20%), Texas (9%), New York (6%), or Georgia (6%). Cases occurred in every month of the year with no seasonal trend observed.

**Table 1.** Demographic Characteristics of Patients With Non-Keratitis *Acanthamoeba* Disease, United States, 1956–2020

Demographic Characteristic	No. (%) (N = 173)
Age, y, median (range) (n = 168)	44 (0–87)
Male sex (n = 171)	121 (71)
Race	
White	52 (30)
Black	21 (12)
Asian/Pacific Islander	2 (1)
American Indian	1 (1)
Other	3 (2)
Unknown	94 (54)
Ethnicity	
Hispanic	17 (10)
Non-Hispanic	23 (13)
Unknown	133 (77)

Data are presented as No. (%) unless otherwise indicated.

Nearly half of patients (47%) had confirmed or suspected involvement of 2 or more organs or organ systems (Table 2). All patients with multiorgan disease had CNS involvement, cutaneous involvement, or both. The most commonly infected organ system was the CNS; 122 patients (71%) were confirmed or suspected to have GAE. Among those, 55% presented with CNS involvement only. Cutaneous disease was confirmed or suspected in 79 patients (46%). Of those, the majority (80%) had multiorgan disease. Likewise, among patients with multiorgan disease, 78% had cutaneous disease. Rhinosinusitis was diagnosed in 51 (29%) patients, 23 patients (13%) had pulmonary involvement, and osteomyelitis was reported in 10 (6%) patients. Most patients with rhinosinusitis, pulmonary disease, and osteomyelitis had multiorgan disease (89%). *Acanthamoeba* was identified in 11 additional organs, most often during postmortem examination. Among 81 patients with multiorgan disease, the most common presentations were GAE with cutaneous disease (17 patients [21%]), and cutaneous disease with rhinosinusitis (17 patients [21%]).

**Table 2.** Clinical Characteristics of Patients With Non-Keratitis *Acanthamoeba* Infections, United States, 1956–2020

Characteristic	Total, No. (%)	Confirmed, No. (%)	Suspected. No. (%)
Organ system involvement^a^			
GAE	122 (71)	105 (61)	17 (10)
Cutaneous	79 (46)	71 (41)	8 (5)
Rhinosinusitis	51 (29)	30 (17)	21 (12)
Pulmonary	23 (13)	17 (10)	6 (3)
Osteomyelitis^b^	10 (6)	8 (5)	2 (1)
Non-keratitis eye disease^c^	5 (3)	2 (1)	3 (2)
Liver	4 (2)	2 (1)	2 (1)
Adrenals	3 (2)	3 (2)	0
Kidney	3 (2)	2 (1)	1 (1)
Lymph nodes	2 (1)	2 (1)	0
Breast	1 (1)	1 (1)	0
Heart	1 (1)	1 (1)	0
Spleen	1 (1)	1 (1)	0
Testicle	1 (1)	1 (1)	0
Thyroid	1 (1)	1 (1)	0
Urethra	1 (1)	1 (1)	0
Total No. of organs infected, confirmed or suspected			
1	92 (53)	…	…
2	51 (29)	…	…
3	18 (10)	…	…
4	6 (3)	…	…
5	4 (2)	…	…
8	2 (1)	…	…
Disease presentation			
CNS involvement			
GAE only	67 (39)	…	…
GAE and cutaneous disease	17 (10)	…	…
GAE and rhinosinusitis	7 (4)	…	…
Other GAE disseminated	31 (18)	…	…
No CNS involvement			
Cutaneous disease only	16 (9)	…	…
Rhinosinusitis only	7 (4)	…	…
Pulmonary only	1 (1)	…	…
Osteomyelitis only	1 (1)	…	…
Cutaneous disease and rhinosinusitis	17 (10)	…	…
Other disseminated (does not include GAE)	9 (5)	…	…
Outcome			
Survived	26 (15)	…	…
Died	122 (71)	…	…
Unknown	25 (14)	…	…
Immunocompromising condition			
HIV infection	67 (39)	…	…
Cancer	48 (28)	…	…
Hematologic malignancy^d^	39 (23)	…	…
Solid organ transplant	31 (18)	…	…
HSCT^e^	18 (10)	…	…
Diabetes mellitus	17 (10)	…	…
Autoimmune disease	14 (8)	…	…
Illicit drug use	11 (6)	…	…
Alcoholism	9 (5)	…	…
Malnourished	2 (1)	…	…
Other immunocompromising condition^f^	5 (3)	…	…
None/unknown	21 (12)	…	…
Exposures			
Soil exposure^g^	19 (11)	…	…
Water exposure^h^	18 (10)	…	…
Nasal irrigation	8 (5)	…	…
Occupational exposure^i^	4 (2)	…	…

Data are presented as No. (%) unless otherwise indicated.

Abbreviations: CNS, central nervous system; GAE, granulomatous amebic encephalitis; HIV, human immunodeficiency virus; HSCT, hematopoietic stem cell transplant.

a Percentages may add up to >100%, as patients could be included in >1 category.

b Many cases of rhinosinusitis described bony erosion into the hard palate on imaging; those cases are not included in the osteomyelitis category.

c Eye disease includes 3 cases of endophthalmitis, 1 case of granulomatous uveitis, and 1 case of iritis.

d Patients with a hematologic malignancy were included in “Cancer” category as well; types of hematologic malignancy included chronic lymphocytic leukemia (13), non-Hodgkin lymphoma (8), acute myeloid leukemia (7), acute lymphocytic leukemia (5), chronic myeloid leukemia (2), Hodgkin lymphoma (2), T-cell prolymphocytic leukemia (1), unspecified lymphoma (1), and unspecified hematologic malignancy (1). One patient had 2 hematologic malignancies.

e Includes bone marrow transplant and stem cell transplant.

f Includes pregnancy, asplenia, primary immunodeficiency, and posttransplant lymphoproliferative disease.

g Soil exposures included gardening (14), farming (4), and other (6), including yard work, mulch exposure, and outdoor recreation. Some patients reported multiple exposures.

h Water exposures included lakes (4), oceans (2), well water (2), swimming pools (6), water park (1), and other (8), including creeks, home medical machines, home fish tanks, and sewage exposure. Some patients reported multiple exposures. This category does not include patients who reported nasal irrigation.

i Occupational exposures included architect, landscaper, gardener, and working with animals.

The majority of patients (88%) had at least 1 immunocompromising condition (Table 2), commonly HIV (39%), cancer (28%), and history of solid organ, bone marrow, or stem cell transplant (28%). Of patients with cancer, 39 (81%) had a hematologic malignancy. Kidneys were the most commonly transplanted organs among solid organ transplant recipients (55%). Availability of environmental exposure data was limited. Among patients with described exposures, the most common were water (10%), soil (11%), and nasal irrigation (5%). All patients reporting nasal irrigation had confirmed or suspected rhinosinusitis (88%) and/or GAE (75%).

Cerebrospinal fluid (CSF) analysis was available for 60 patients with confirmed or suspected GAE (49%) (Table 3). The median protein was 99 mg/dL, median glucose was 59 mg/dL, and median opening pressure was 200 mm H_2_O. The median white blood cell count was 29 cells/μL, ranging from 0 to 2384 cells/μL. Among samples with pleocytosis, some exhibited lymphocyte predominance, whereas others exhibited neutrophil predominance. *Acanthamoeba* was identified in the CSF by PCR in 8 patients, though the total number tested is unknown.

**Table 3.** Cerebrospinal Fluid Findings Among Patients With *Acanthamoeba* Granulomatous Amebic Encephalitis, United States, 1956–2020

CSF Finding	No. of Patients With GAE With Available Data (n = 60)	Median (IQR)	Range
WBC count, cells/µL	59	29 (2–150)	0–2384
Neutrophils, %	34	28 (8–73)	0–88
Monocytes, %	34	12 (4–26)	0–90
Lymphocytes, %	39	43 (7–80)	0–98
RBC count, cells/µL	51	5 (0–66)	0–8450
Protein, mg/dL	54	99 (59–176)	11–901
Glucose, mg/dL	54	59 (45–88)	11–150
Opening pressure, mm H_2_O	14	200 (90–250)	18–550

Of those with confirmed or suspected GAE (n = 122), 60 (49%) had known CSF results.

Abbreviations: CSF, cerebrospinal fluid; GAE, granulomatous amebic encephalitis; IQR, interquartile range; RBC, red blood cell; WBC, white blood cell.

Genotype results were available for 36 cases. Genotypes T4 and T1 were most common (18 [50%] and 14 [39%], respectively; Table 4). Among those with T4 genotype, 11 patients (61%) had confirmed or suspected GAE, 12 (67%) had skin involvement, and 6 (33%) had rhinosinusitis. All 14 patients with T1 genotype had confirmed GAE (100%), 2 (14%) had skin involvement, and 2 (14%) had rhinosinusitis. More patients with T1 genotype had single-organ disease (11 [79%]) compared with T4 genotype (7 [39%]).

**Table 4.** Association Between Demographic and Clinical Characteristics and Survival of Patients With Non-Keratitis *Acanthamoeba* Infections, United States, 1956–2020

Characteristic	Total (n = 148)^a^	Survived (n = 26 [18%])	Died (n = 122 [82%])	χ^2^*P* Value
	No. (Column %)	No. (Row %)	No. (Row %)	
Age, y				.4785^b^
0–30	27 (18)	2 (7)	25 (93)	
31–50	59 (40)	12 (20)	47 (80)	
51–64	40 (27)	8 (20)	32 (80)	
≥65	21 (14)	4 (19)	17 (81)	
Sex				.**0167**
Male	103 (70)	13 (13)	90 (87)	
Female	45 (30)	13 (29)	32 (71)	
Race				.2786^b^
White	45 (30)	10 (22)	35 (78)	
Black	19 (13)	4 (21)	15 (79)	
Other	6 (4)	2 (33)	4 (67)	
Unknown	78 (53)	10 (13)	68 (87)	
Ethnicity				.1500^b^
Hispanic	16 (11)	3 (19)	13 (81)	
Non-Hispanic	22 (15)	7 (32)	15 (68)	
Unknown	110 (74)	16 (15)	94 (85)	
Total No. of organ systems infected, confirmed or suspected				.3472
1	75 (51)	11 (15)	64 (85)	
≥ 2	73 (49)	15 (21)	58 (79)	
Disease presentation (n = 147)^c^				**<**.**0001^b^**
CNS involvement				
GAE only	59 (40)	2 (3)	57 (97)	
GAE and cutaneous disease	15 (10)	2 (13)	13 (87)	
GAE and rhinosinusitis	7 (5)	1 (14)	6 (86)	
Other GAE disseminated	31 (21)	2 (6)	29 (94)	
No CNS involvement				
Cutaneous disease only	12 (8)	6 (50)	6 (50)	
Rhinosinusitis only	3 (2)	3 (100)	0 (0)	
Cutaneous disease and rhinosinusitis	11 (7)	3 (27)	8 (73)	
Other disseminated (does not include GAE)	9 (6)	7 (78)	2 (22)	
Immunocompromising condition				
Cancer	39 (26)	8 (21)	31 (79)	.5733
No	109 (74)	18 (17)	91 (83)	
Solid organ transplant	29 (20)	9 (31)	20 (69)	.**0336**
No	119 (80)	17 (14)	102 (86)	
Stem cell transplant	16 (11)	3 (19)	13 (81)	1^b^
No	132 (89)	23 (17)	109 (83)	
HIV infection	58 (39)	8 (14)	50 (86)	.3327
No	90 (61)	18 (20)	72 (80)	
Autoimmune disease	12 (8)	3 (25)	9 (75)	.4427^b^
No	136 (92)	23 (17)	113 (83)	
Diabetes mellitus	15 (10)	5 (33)	10 (67)	.0905
No	133 (90)	21 (16)	112 (84)	
None/unknown	15 (10)	0 (0)	15 (100)	.0593
Immunocompromised	133 (90)	26 (20)	107 (80)	
Year				.0503^b^
1956–1979	11 (7)	1 (9)	10 (91)	
1980–1989	11 (7)	0 (0)	11 (100)	
1990–1999	43 (29)	4 (9)	39 (91)	
2000–2009	28 (19)	5 (18)	23 (82)	
2010–2020	55 (37)	16 (29)	39 (71)	
Genotype^d^				.1608
T1	14 (10)	2 (14)	12 (86)	
T4	16 (11)	8 (50)	8 (50)	
T5	1 (1)	0 (0)	1 (100)	
T17	1 (1)	0 (0)	1 (100)	
T18	2 (1)	0 (0)	2 (100)	
Treatments (n = 72)^e^				
Miltefosine	21 (29)	10 (48)	11 (52)	.0989
No/unknown	51 (71)	14 (27)	37 (73)	
Pentamidine	28 (39)	12 (43)	16 (57)	.1715
No/unknown	44 (61)	12 (27)	32 (73)	
Sulfadiazine	20 (28)	8 (40)	12 (60)	.4568
No/unknown	52 (72)	16 (31)	36 (69)	
Flucytosine	25 (35)	14 (56)	11 (44)	.**0029**
No/unknown	47 (65)	10 (21)	37 (79)	
Fluconazole	27 (38)	9 (33)	18 (67)	1
No/unknown	45 (63)	15 (33)	30 (67)	
Currently recommended regimen^f^	7 (10)	5 (71)	2 (29)	.**0372^b^**
No/unknown	65 (90)	19 (29)	46 (71)	
Any triazole (fluconazole, itraconazole, or voriconazole)	44 (61)	19 (44)	25 (57)	.**0263**
No/unknown	28 (39)	5 (18)	23 (82)	

Bold indicates statistical significance.

Abbreviations: CNS, central nervous system; GAE, granulomatous amebic encephalitis; HIV, human immunodeficiency virus.

a Excluded 25 patients with unknown survival outcome.

b Fisher exact test used instead of χ^2^ when >25% of cells had values <5.

c n = 147 (1 additional patient excluded with isolated pulmonary disease in setting of lung transplantation).

d n = 34 patients with known genotype.

e n = 72 patients with antemortem diagnosis.

f Recommended regimen: miltefosine + pentamidine + sulfadiazine + flucytosine + fluconazole.

Of those with known outcomes, 26 patients (18%) survived (Table 4). A higher proportion of female patients survived compared with males (29% and 13%, respectively; *P* = .0167). No significant association was found between survival and age, race, or ethnicity. The 26 survivors were immunocompromised; all 15 patients who did not have immunocompromising conditions or had unknown immunostatus died. Among 8 survivors with cancer, 5 had chronic lymphocytic leukemia. Patients with GAE, either isolated or with multiorgan disease, had higher mortality compared with patients without GAE (*P* < .0001). The odds of survival were 17.8 times higher among patients without GAE compared to those with GAE (95% confidence interval, 6.5–49.1). Survival has increased over time. More patients diagnosed in the most recent decade (2010–2020) survived than patients in previous decades (29% vs 0%–18%, respectively; *P* = .0503). Among the 16 cases with T4 genotype for which patient outcome was known, 50% survived, compared with 14% among those with the T1 genotype.

Among 72 patients with antemortem *Acanthamoeba* diagnoses, there was a significant association between survival and receiving the currently recommended treatment regimen. Five of 7 patients who received pentamidine, fluconazole, flucytosine, miltefosine, and sulfadiazine survived (*P* = .0372; Table 4). Most patients (61%) received at least 1 triazole (fluconazole, itraconazole, or voriconazole), which was also associated with survival (*P* = .0263).

## DISCUSSION

This comprehensive case series of non-keratitis *Acanthamoeba* infections in the US reinforces that *Acanthamoeba* infections are rare, can involve multiple organ systems, and are often fatal. While *Acanthamoeba* is typically thought to cause keratitis, GAE, or cutaneous disease, this report highlights the importance of recognizing other possible manifestations of *Acanthamoeba* infections as well, including rhinosinusitis, pulmonary disease, osteomyelitis, and multiorgan disease. While survival is infrequent, the prognosis is better for patients without CNS involvement.

Prior to this report, *Acanthamoeba* infections in the literature have often been categorized as GAE, cutaneous, rhinosinusitis, and disseminated. The data presented here suggest that these categories may not be capturing the variety of ways *Acanthamoeba* can spread throughout the body. Among the 173 patients identified in this study, nearly half had involvement of >1 organ system, and there were 33 different combinations of organ systems involved. This number of disease presentations underscores that disseminated disease can look very different among different patients.

Despite this variability, the majority of patients had CNS involvement. Among cases that did not involve the CNS, nearly all involved either the skin or the sinuses. This supports the theory that infection is acquired through direct inoculation (eg, skin and sinus disease) or through hematogenous spread, in cases of CNS and multiorgan disease [28]. Although CNS disease was the most common presentation, skin was the most frequent organ affected in patients with multiorgan disease. Clinicians should be aware that *Acanthamoeba* can present with involvement beyond the CNS and skin.

This case series underscores the frequent mortality associated with non-keratitis *Acanthamoeba* infections and identifies characteristics that may be associated with survival. The characteristic most often associated with survival was absence of CNS disease. In fact, the odds of survival were nearly 18 times higher in patients without GAE compared to those with GAE. This finding emphasizes the importance of identifying, diagnosing, and treating *Acanthamoeba* cutaneous disease and rhinosinusitis as early in the disease course as possible so that dissemination to the CNS can be avoided. It also highlights the importance of considering the diagnosis of GAE in a patient presenting with unexplained encephalitis, especially if immunocompromised.

While patients with encephalitis often undergo CSF examination, CSF findings for *Acanthamoeba* are not particularly helpful in the diagnosis. Many patients with GAE had increased CSF protein level and pleocytosis, though not all. Several patients with confirmed GAE in this case series had normal CSF profiles, underscoring the importance of tissue biopsy if clinical suspicion for *Acanthamoeba* is high.

In the 1980s and 1990s, most invasive *Acanthamoeba* infections were diagnosed in people living with HIV (PLWH) [12, 13, 25, 29–34]. However, fewer PLWH have been diagnosed with *Acanthamoeba* in recent years, likely due to developments in HIV treatments and improved immune status. All PLWH included in this analysis over the past 10 years have had CD4 counts <100 cells/µL, suggesting that PLWH with adequately controlled HIV are at lower risk. In the past 2 decades, most cases of *Acanthamoeba* have occurred among people with cancer or who have undergone solid organ or hematopoietic stem cell transplant, likely due to the profound level of immunosuppression induced by chemotherapy and other medications [14, 35–37]. There is an unexplained predominance of cases among people with hematologic malignancies, especially leukemia, which may be due to unique immunological deficits among these patients, either because of their primary disease or medications used for treatment. Interestingly, all patients who either had no immunocompromising condition or whose immune status was unknown died. Although the implications of this finding are unclear, it is possibly due to missing information. Many of these cases occurred in the 1970s and 1980s, when surveillance data were often incomplete. While it is possible that these cases occurred in healthy individuals, it is more likely that their full medical histories were not reported. It is also possible that the diagnosis was delayed due to later presentation to medical care or lower level of clinical suspicion compared to those with immunocompromising conditions.

The geographic and seasonal distribution of invasive *Acanthamoeba* infections indicate that these infections occur year-round throughout the US. This differs from *N fowleri*, which typically infects humans during warm months and in states with warmer climates through nasal exposure to water [9, 38]. Although the source of exposure is often unknown for *Acanthamoeba* cases, these amebae have been isolated from water sources in homes as well as air conditioning units and ventilation ducts [1]. In this case series, we identified 8 patients who reported nasal rinsing. Using unsterile water for nasal rinsing increases the risk of many infections, including *Acanthamoeba* and other FLA [39, 40]. Patients who perform nasal rinsing, especially if immunocompromised, should be counseled about how to minimize their risk of infection by using sterile water for both rinsing and cleaning equipment [41].

This analysis suggests that males are more likely to be diagnosed with non-keratitis *Acanthamoeba* infections and also have a lower rate of survival compared with females. Infections with other FLA are also more common among males, and it has been suggested that increased risk behaviors such as participating in water activities or occupational soil exposure may be contributing factors [8, 9, 42]. These exposures are less likely to be contributing to *Acanthamoeba* infections, given that these environmental risk factors are not as prevalent among cases. Other reasons for this discrepancy could be related to differences in predisposing medical conditions. For example, in 2018, 81% of new HIV diagnoses in the US occurred in men [43]. Similarly, the incidence of cancer is higher in men, including hematologic malignancies like leukemia [44]. Among those with recorded race, 27% were Black, which is higher than the expected race distribution according to US census data. This is possibly due to racial disparities, as some medical conditions are more prevalent among Black patients including HIV, cancer, diabetes, end-stage renal disease, and other conditions that that constitute a risk for *Acanthamoeba* infection [44, 45]. In this analysis, most patients (77%) did not have a recorded ethnicity. However, among those with known ethnicity, Hispanic people represented a large proportion (43%). Similar findings have been reported for *Balamuthia mandrillaris* infections [8]. It is unclear whether this finding is an artifact of missing data using an unrepresentative sample, or whether there may truly be an association between ethnicity and prevalence of *Acanthamoeba* infections.

*Acanthamoeba* is currently classified into 23 known genotype groups by molecular sequencing (T1–T23), but not all have been associated with human disease [46–48]. Genotype T4 has been most frequently identified in human infections and is thought to be the most prevalent in the environment, often causing cases of *Acanthamoeba* keratitis and GAE [15, 46, 47, 49, 50]. While genotype T1 has been identified in human non-keratitis *Acanthamoeba* infections previously, it does not have the same environmental ubiquity as genotype T4 [46]. In addition to disease severity, genotype may also be associated with the organ system(s) involved. In this analysis, all patients known to have T1 genotype had confirmed GAE; however, cutaneous disease and rhinosinusitis were more common among those known to have T1 genotype. As such, more cases involving T4 genotype survived. Although the sample size for the genotype analysis was small, it is possible that this trend may be indicative of differences in virulence or pathogenicity among different genotypes of *Acanthamoeba*.

Both case counts and patient survival have increased over time, likely due to improved surveillance, provider awareness, diagnostic abilities, and treatment options. CDC's Free-Living and Intestinal Ameba Laboratory was established in the early 1970s, shortly after the discovery of FLA as pathogens. As awareness of FLA increased and more diagnostic techniques became available, more cases were identified. CDC developed a clinical consultation service in 2010 to assist clinicians with diagnosis and treatment of FLA. These efforts may have also increased the number of cases diagnosed and reported to CDC. Over time, more was learned about treatment options for *Acanthamoeba* infections, which may have contributed to improved patient outcomes. In this analysis, the most commonly prescribed medications among patients with antemortem *Acanthamoeba* diagnoses included those currently recommended for treatment—miltefosine, flucytosine, fluconazole, pentamidine, and sulfadiazine—as well as several other antimicrobial medications. The recommended regimen was associated with survival in a bivariate analysis, though sample size was small. Triazole use was also independently found to be associated with survival. This finding supports prioritizing a triazole in the regimen when possible.

The major limitation of this study is missing and incomplete data. Also, some cases might not have been reported to CDC or to state health departments since *Acanthamoeba* infection is not a nationally notifiable condition nor reportable in most states. It is also likely that many *Acanthamoeba* infections remain undiagnosed due to limited awareness and access to diagnostic capabilities. *Acanthamoeba* should be considered for any nonhealing skin lesion, persistent rhinosinusitis, or unexplained CNS symptoms in an immunocompromised individual. Early recognition and diagnosis could improve patient outcomes, as treatment has been associated with survival in patients with non-keratitis *Acanthamoeba* infections. CDC's FLA consultation service is available 24/7 to provide diagnostic and clinical assistance to clinicians caring for patients with confirmed or suspected *Acanthamoeba* infections. Clinicians should call the CDC Emergency Operations Center at (770) 488-7100 if a consultation is desired and should notify either their local or state health department or CDC of laboratory-confirmed cases.

## Notes

***Acknowledgments.*** The authors acknowledge Dr Govinda Visvesvara who established the free-living ameba laboratory at the Centers for Disease Control and Prevention (CDC), and without whom this work would not have been possible. Also, we thank Megan Gerdes for statistical support, as well as our public health partners who have reported cases over the years.

***Financial support*.** The authors have no financial support to disclose.

***Patient consent*.** As this activity involves a public health authority testing information to assess the patterns and risk factors associated with a condition of public health importance, it is consistent with nonresearch public health surveillance. This work conforms to ethical standards applied in the United States by the CDC, as described in the Methods section of the manuscript.

## References

[1] Visvesvara GS , Moura H, Schuster FL. Pathogenic and opportunistic free-living amoebae: *Acanthamoeba* spp., *Balamuthia mandrillaris*, *Naegleria fowleri*, and *Sappinia diploidea*. FEMS Immunol Med Microbiol 2007; 50:1–26. 17428307 10.1111/j.1574-695X.2007.00232.x

[2] Martinez AJ , Visvesvara GS. Free-living, amphizoic and opportunistic amebas. Brain Pathol 1997; 7:583–98. 9034567 10.1111/j.1750-3639.1997.tb01076.x PMC8098488

[3] Stockman LJ , Wright CJ, Visvesvara GS, Fields BS, Beach MJ. Prevalence of *Acanthamoeba* spp. and other free-living amoebae in household water, Ohio, USA-1990–1992. Parasitol Res 2011; 108:621–7. 20978791 10.1007/s00436-010-2120-7

[4] Jager BV , Stamm WP. Brain abscesses caused by free-living amoeba probably of the genus *Hartmannella* in a patient with Hodgkin’s disease. Lancet 1972; 2:1343–5. 4118209 10.1016/s0140-6736(72)92781-x

[5] Martinez AJ . Is *Acanthamoeba* encephalitis an opportunistic infection? Neurology 1980; 30:567–74. 6991973 10.1212/wnl.30.6.567

[6] Kernohan JW , Magath TB, Schloss GT. Granuloma of brain probably due to *Endolimax williamsi* (*Iodamoeba butschlii*). Arch Pathol 1960; 70:576–80. 13752647

[7] Martinez AJ . Acanthamoebiasis and immunosuppression: case report. J Neuropathol Exp Neurol 1982; 41:548–57. 7108568 10.1097/00005072-198209000-00007

[8] Cope JR , Landa J, Nethercut H, et al The epidemiology and clinical features of *Balamuthia mandrillaris* disease in the United States, 1974–2016. Clin Infect Dis 2019; 68:1815–22. 30239654 10.1093/cid/ciy813 PMC7453664

[9] Yoder JS , Eddy BA, Visvesvara GS, Capewell L, Beach MJ. The epidemiology of primary amoebic meningoencephalitis in the USA, 1962–2008. Epidemiol Infect 2010; 138:968–75. 19845995 10.1017/S0950268809991014

[10] Martinez AJ , Janitschke K. *Acanthamoeba*, an opportunistic microorganism: a review. Infection 1985; 13:251–6. 2867047 10.1007/BF01645432

[11] Schuster FL , Visvesvara GS. Free-living amoebae as opportunistic and non-opportunistic pathogens of humans and animals. Int J Parasitol 2004; 34:1001–27. 15313128 10.1016/j.ijpara.2004.06.004

[12] Gonzalez MM , Gould E, Dickinson G, et al Acquired immunodeficiency syndrome associated with *Acanthamoeba* infection and other opportunistic organisms. Arch Pathol Lab Med 1986; 110:749–51. 3488048

[13] Murakawa GJ , McCalmont T, Altman J, et al Disseminated acanthamebiasis in patients with AIDS: a report of five cases and a review of the literature. Arch Dermatol 1995; 131:1291–6. 7503573

[14] Satlin MJ , Graham JK, Visvesvara GS, et al Fulminant and fatal encephalitis caused by *Acanthamoeba* in a kidney transplant recipient: case report and literature review. Transpl Infect Dis 2013; 15:619–26. 24010955 10.1111/tid.12131

[15] Khan NA . *Acanthamoeba*: biology and increasing importance in human health. FEMS Microbiol Rev 2006; 30:564–95. 16774587 10.1111/j.1574-6976.2006.00023.x

[16] Damhorst GL , Watts A, Hernandez-Romieu A, et al *Acanthamoeba castellanii* encephalitis in a patient with AIDS: a case report and literature review. Lancet Infect Dis 2021; 22:e59–65. 34461057 10.1016/S1473-3099(20)30933-6 PMC10910629

[17] Visvesvara GS , Stehr-Green JK. Epidemiology of free-living ameba infections. J Protozool 1990; 37:25S–33S. 2258827 10.1111/j.1550-7408.1990.tb01142.x

[18] Paltiel M , Powell E, Lynch J, Baranowski B, Martins C. Disseminated cutaneous acanthamebiasis: a case report and review of the literature. Cutis 2004; 73:241–8. 15134324

[19] Galarza C , Ramos W, Gutierrez EL, et al Cutaneous acanthamebiasis infection in immunocompetent and immunocompromised patients. Int J Dermatol 2009; 48:1324–9. 20415673 10.1111/j.1365-4632.2008.03786.x

[20] Teknos TN , Poulin MD, Laruentano AM, Li KK. *Acanthamoeba rhinosinusitis*: characterization, diagnosis, and treatment. Am J Rhinol 2000; 14:387–91. 11197115 10.2500/105065800779954293

[21] Vernon SE , Acar BC, Pham SM, Fertel D. *Acanthamoeba* infection in lung transplantation: report of a case and review of the literature. Transpl Infect Dis 2005; 7:154–7. 16390406 10.1111/j.1399-3062.2005.00113.x

[22] Steinberg JP , Galindo RL, Kraus ES, Ghanem KG. Disseminated acanthamebiasis in a renal transplant recipient with osteomyelitis and cutaneous lesions: case report and literature review. Clin Infect Dis 2002; 35:e43–9. 12173148 10.1086/341973

[23] Selby DM , Chandra RS, Rakusan TA, Loechelt B, Markle BM, Visvesvara GS. Amebic osteomyelitis in a child with acquired immunodeficiency syndrome: a case report. Pediatr Pathol Lab Med 1998; 18:89–95. 9566286

[24] Duarte AG , Sattar F, Granwehr B, Aronson JF, Wang Z, Lick S. Disseminated acanthamoebiasis after lung transplantation. J Heart Lung Transplant 2006; 25:237–40. 16446227 10.1016/j.healun.2005.09.006

[25] Sison JP , Kemper CA, Loveless M, McShane D, Visvesvara GS, Deresinski SC. Disseminated *Acanthamoeba* infection in patients with AIDS: case reports and review. Clin Infect Dis 1995; 20:1207–16. 7620001 10.1093/clinids/20.5.1207

[26] Young AL , Leboeuf NR, Tsiouris SJ, Husain S, Grossman ME. Fatal disseminated *Acanthamoeba* infection in a liver transplant recipient immunocompromised by combination therapies for graft-versus-host disease. Transpl Infect Dis 2010; 12:529–37. 20604904 10.1111/j.1399-3062.2010.00535.x

[27] Centers for Disease Control and Prevention . Free-living amebae infections 2012 case definition. 2021.https://ndc.services.cdc.gov/case-definitions/free-living-amebae-infections-2012/. Accessed April 29, 2022.

[28] Centers for Disease Control and Prevention . DPDx—free living amebic infections. 2019.https://www.cdc.gov/dpdx/freelivingamebic/index.html. Accessed April 29, 2022.

[29] Wiley CA , Safrin RE, Davis CE, et al *Acanthamoeba* meningoencephalitis in a patient with AIDS. J Infect Dis 1987; 155:130–3. 3794397 10.1093/infdis/155.1.130

[30] Gardner HA , Martinez AJ, Visvesvara GS, Sotrel A. Granulomatous amebic encephalitis in an AIDS patient. Neurology 1991; 41:1993–5. 1745363 10.1212/wnl.41.12.1993

[31] Friedland LR , Raphael SA, Deutsch ES, et al Disseminated *Acanthamoeba* infection in a child with symptomatic human immunodeficiency virus infection. Pediatr Infect Dis J 1992; 11:404–7. 1630862 10.1097/00006454-199205000-00012

[32] Gordon SM , Steinberg JP, DuPuis MH, Kozarsky PE, Nickerson JF, Visvesvara GS. Culture isolation of *Acanthamoeba* species and leptomyxid amebas from patients with amebic meningoencephalitis, including two patients with AIDS. Clin Infect Dis 1992; 15:1024–30. 1457633 10.1093/clind/15.6.1024

[33] May LP , Sidhu GS, Buchness MR. Diagnosis of *Acanthamoeba* infection by cutaneous manifestations in a man seropositive to HIV. J Am Acad Dermatol 1992; 26(2 Pt 2):352–5. 1569257 10.1016/0190-9622(92)70054-j

[34] Tan B , Weldon-Linne CM, Rhone DP, Penning CL, Visvesvara GS. *Acanthamoeba* infection presenting as skin lesions in patients with the acquired immunodeficiency syndrome. Arch Pathol Lab Med 1993; 117:1043–6. 8215828

[35] Keane NA , Lane LM, Canniff E, et al A surviving case of *Acanthamoeba* granulomatous amebic encephalitis in a hematopoietic stem cell transplant recipient. Am J Case Rep 2020; 21:e923219. 10.12659/AJCR.923219 PMC7347033 32603318

[36] Winsett F , Dietert J, Tschen J, Swaby M, Bangert CA. A rare case of cutaneous acanthamoebiasis in a renal transplant patient. Dermatol Online J 2017; 23:13030/qt88s2t7wp. 28329521

[37] Castellano-Sanchez A , Popp AC, Nolte FS, et al *Acanthamoeba castellani* encephalitis following partially mismatched related donor peripheral stem cell transplantation. Transpl Infect Dis 2003; 5:191–4. 14987204 10.1111/j.1399-3062.2003.00029.x

[38] Gharpure R , Gleason M, Salah Z, et al Geographic range of recreational water-associated primary amebic meningoencephalitis, United States, 1978–2018. Emerg Infect Dis 2021; 27:271–4. 33350926 10.3201/eid2701.202119 PMC7774533

[39] Cope JR , Roy S, Ali I. *Acanthamoeba* disease associated with the practice of nasal rinsing in immunocompromised patients. Open Forum Infect Dis 2018; 5:S22.

[40] Sazzad HMS , Luby SP, Sejvar J, et al A case of primary amebic meningoencephalitis caused by *Naegleria fowleri* in Bangladesh. Parasitol Res 2020; 119:339–44. 31734864 10.1007/s00436-019-06463-y

[41] Centers for Disease Control and Prevention . Sinus rinsing for health or religious practice. 2017.https://www.cdc.gov/parasites/naegleria/sinus-rinsing.html. Accessed April 29, 2022.

[42] Gharpure R , Bliton J, Goodman A, Ali IKM, Yoder J, Cope JR. Epidemiology and clinical characteristics of primary amebic meningoencephalitis caused by *Naegleria fowleri*: a global review. Clin Infect Dis 2021; 73:e19–27. 32369575 10.1093/cid/ciaa520 PMC8739754

[43] Centers for Disease Control and Prevention . HIV and men. 2022.https://www.cdc.gov/hiv/group/gender/men/index.html. Accessed April 29, 2022.

[44] Siegel RL , Miller KD, Fuchs HE, Jemal A. Cancer statistics, 2021. CA Cancer J Clin 2021; 71:7–33. 33433946 10.3322/caac.21654

[45] Centers for Disease Control and Prevention . HIV and African American people: HIV incidence. 2022.https://www.cdc.gov/hiv/group/racialethnic/africanamericans/incidence.html. Accessed April 29, 2022.

[46] Booton GC , Visvesvara GS, Byers TJ, Kelly DJ, Fuerst PA. Identification and distribution of *Acanthamoeba* species genotypes associated with nonkeratitis infections. J Clin Microbiol 2005; 43:1689–93. 15814986 10.1128/JCM.43.4.1689-1693.2005 PMC1081337

[47] Diehl MLN , Paes J, Rott MB. Genotype distribution of *Acanthamoeba* in keratitis: a systematic review. Parasitol Res 2021; 120:3051–63. 34351492 10.1007/s00436-021-07261-1 PMC8339388

[48] Putaporntip C , Kuamsab N, Nuprasert W. Genotypes from public freshwater sources in Thailand reveals a new genotype, T23 *Acanthamoeba bangkokensis* sp. nov. Sci Rep 2021; 11:17290. 34453084 10.1038/s41598-021-96690-0 PMC8397737

[49] Maciver SK , Asif M, Simmen MW, Lorenzo-Morales J. A systematic analysis of *Acanthamoeba* genotype frequency correlated with source and pathogenicity: T4 is confirmed as a pathogen-rich genotype. Eur J Protistol 2013; 49:217–21. 23290304 10.1016/j.ejop.2012.11.004

[50] Behera HS , Satpathy G, Tripathi M. Isolation and genotyping of *Acanthamoeba* spp. from *Acanthamoeba* meningitis/meningoencephalitis (AME) patients in India. Parasit Vectors 2016; 9:442. 27507421 10.1186/s13071-016-1729-5 PMC4977702

